# Tocotrienols Ameliorate Neurodegeneration and Motor Deficits in the 6-OHDA-Induced Rat Model of Parkinsonism: Behavioural and Immunohistochemistry Analysis

**DOI:** 10.3390/nu13051583

**Published:** 2021-05-10

**Authors:** Mangala Kumari, Premdass Ramdas, Ammu Kutty Radhakrishnan, Methil Kannan Kutty, Nagaraja Haleagrahara

**Affiliations:** 1 Department of Anatomy, Division of Human Biology, School of Medicine, International Medical University, Kuala Lumpur 57000, Malaysia; 2 Division of Applied Biomedical Sciences and Biotechnology, School of Health Sciences, International Medical University, Kuala Lumpur 57000, Malaysia; premdass_ramdas@imu.edu.my; 3 Jeffrey Cheah School of Medicine and Health Sciences, Monash University Malaysia, Bandar Sunway, Selangor 47500, Malaysia; Ammu.Radhakrishnan@monash.edu; 4 Department of Medicine, Lincoln University College, Kelana Jaya, Selangor 47301, Malaysia; methilkannan@gmail.com; 5 College of Medicine and Dentistry, James Cook University, Townsville, QLD 4811, Australia; haleagrahara.nagaraja@jcu.edu.au

**Keywords:** vitamin E, tocotrienols, tyrosine hydroxylase, Parkinson’s disease, neurofunctions, neuroglia, neurofilaments, glial fibrillary acidic protein

## Abstract

Parkinson’s disease (PD) is a debilitating neurodegenerative disease, which progresses over time, causing pathological depigmentation of the substantia nigra (SN) in the midbrain due to loss of dopaminergic neurons. Emerging studies revealed the promising effects of some nutrient compounds in reducing the risk of PD. One such nutrient compound that possess neuroprotective effects and prevents neurodegeneration is tocotrienol (T3), a vitamin E family member. In the present study, a single dose intracisternal injection of 250 µg 6-hydroxydopamine (6-OHDA) was used to induce parkinsonism in male Sprague Dawley (SD) rats. Forty-eight hours post injection, the SD rats were orally supplemented with alpha (α)- and gamma (γ)-T3 for 28 days. The neuroprotective effects of α- and γ-T3 were evaluated using behavioural studies and immunohistochemistry (IHC). The findings from this study revealed that supplementation of α- and γ-T3 was able to ameliorate the motor deficits induced by 6-OHDA and improve the neuronal functions by reducing inflammation, reversing the neuronal degradation, and preventing further reduction of dopaminergic neurons in the SN and striatum (STR) fibre density.

## 1. Introduction

Parkinson’s disease (PD) is the second most common progressive neurodegenerative disease in humans after Alzheimer’s disease [1]. The number of people with PD is estimated to be between 4 to 6 million in the most populous nations. Longitudinal meta-analysis data showed that the prevalence of PD steadily increased with age. Besides, the geographical prevalence is 2.5 times higher in people from Australia, Europe, and America compared to the individuals from Asia [2]. 

The selective loss of dopaminergic neurons in the pars compacta region of the substantia nigra (SN) of the midbrain and reduced striatal dopamine (DA) levels in the brain are the main pathological features that lead to the development of PD. These pathologies cause disturbances in the nigrostriatal dopaminergic pathway, which result in significant clinical abnormalities that manifest as motor and non-motor symptoms [3]. The motor symptoms of PD include tremor at rest, muscle rigidity, bradykinesia, postural instability, and autonomic disturbances. Some of the non-motor symptoms of PD include sleep disturbance, cognitive-related deficits, anosmia, depression, and gastrointestinal disorders [4].

Neuroinflammation induced by activated neuroglia is also linked to the pathogenesis of PD. Activated neuroglia secretes pro-inflammatory cytokines, which further aggravate the disease [5]. Researchers postulated that drugs that target activated neuroglia and reduce the levels of pro-inflammatory cytokines in the brain are suitable candidate drug(s) for PD, as these types of drugs can reduce neuroinflammation and also alleviate neuronal oxidative stress [6]. Interestingly, some nutrients have been known to target activated neuroglia and have been reported to have profound effects in regulating the proinflammatory cytokines. Studies have revealed the role of some nutrients in reducing the risk of PD by preventing neurodegeneration or halting the disease progression [7]. 

One such natural compound is tocotrienols (T3), a vitamin E family member, which is a naturally occurring fat-soluble vitamin [8]. Tocotrienols are found naturally in annatto seeds, rice bran, and particularly palm oil and rice bran oil in which higher amounts of tocotrienols are present [9]. Several studies have shown that T3 possesses many health-enhancing effects such as anti-cancer [10,11], anti-proliferative [12,13], anti-thrombotic [14], anti-inflammatory [15], antioxidant [16], and neuroprotective [17,18] effects. Palm oil-derived tocotrienol-rich fraction (TRF) has been reported to provide immense protection against cytotoxicity caused by oxidative stress (OS) in rat striatal cells [19]. A recent systematic review on the safety and neuroprotective efficacy of palm-oil and TRF concluded that T3 could enhance the healthy animals’ cognitive function and attenuate OS [20]. According to Khanna et al., α-T3 exhibits cellular protection of the nerves through two main pathways: 12-lipoxygenase (LOX) and c-Src pathways [21]. However, the reports on the neuroprotective effects of γ-T3 are minimal, in particular, the effects of γ-T3 supplementation on the 6-OHDA-induced rat model of parkinsonism are not available in the literature. A previous clinical trial showed the protective effects of mixed T3 in decreasing the progression of white matter lesions (WML) in 121 human volunteers [22]. Moreover, palm vitamin E has achieved the “generally regarded as safe (GRAS)” status by the Food and Drug Administration (FDA), USA [23]. It is considered safe for consumption and has been attributed with many health-enhancing effects including brain health [24]. 

Animal models using various types of neurotoxins are popular tools that have been used for the study of PD over the past couple of decades and have imparted significant knowledge on the aetiology and pathogenesis of PD [25]. The specific target of neurotoxins in the animal models is the catecholaminergic neurons, which induce PD-related symptoms and pathology when impaired by neurotoxins. The neurotoxin 6-OHDA cannot cross the blood–brain barrier; hence, systemic administration will not cause parkinsonism [26]. The degree of the lesion with 6-OHDA depends on the route of administration (intracerebral or intracisternal), concentration, and the species used [27,28,29]. Hence, this study aimed to evaluate the neuroprotective efficacy of oral supplementation of isomers of T3 (α- and γ-T3) on the intracisternal 6-OHDA-injected rat model. With this study, we sought to determine whether isomers of T3 (α- and γ-T3) could reverse parkinsonian motor symptoms on 6-OHDA-injected SD rats and prevent the DA neuronal loss in agreement with the behavioural observation of the corresponding animals. 

## 2. Materials and Methods

### 2.1. Induction of Parkinsonism and Tocotrienol Isomers (α- and γ-T3) Supplementation

In this study, twelve to fourteen-week-old male Sprague Dawley (SD) rats (weighing 200–250 g) were allowed to acclimatise for one week in the animal holding facility (AHF) at the International Medical University (IMU, Malaysia), with a climate-controlled (25 ± 2 °C) environment and 12-h light/dark cycle. Following this, the SD rats were injected intracisternally with 10 µl of freshly prepared 6-OHDA solution containing 250 µg of 6-OHDA to induce parkinsonism as described previously [29]. Injected SD rats were monitored for 48 h and assigned into three groups; (i) 6-OHDA injected, (ii) 6-ODHA injected + α-T3 supplemented, and (iii) 6-ODHA injected + γ-T3 supplemented. Each group had six rats per group. A group of rats (*n* = 6) that were not injected with 6-OHDA served as control/baseline.

Two isomers of T3, namely, α- and γ-T3 used for oral supplementation, were at a purity of approximately 97%, a kind gift from Davos Life Science Pvt. Ltd. (Singapore). These treatments were freshly prepared daily for oral feeding of SD rats induced with 6-OHDA. The SD rats in the treatment groups were fed with 10 mg/kg body weight (bw) of T3 (α- or γ-T3) [30] for 28 days through oral gavage. 

### 2.2. Behavioural Evaluation

The SD rats were subjected to pre-test training on all the behavioural parameters with three trials each from day 4 to day 7, during the acclimatisation period. However, the data of the pre-test training was not collected as the purpose of this was to facilitate animal learning and adaptation to the behavioural parameters. The apparatus was cleaned after each test with an aqueous solution containing ethanol (5% *v/v*) to avoid possible biasing effect from the previous rats’ odour cues between trials. The subsequent tests were performed on day 7, day 14, day 21, and day 28 after the supplementation with T3. The mean of the three trials for each of the tests was calculated as the individual score.

#### 2.2.1. Paw Retraction Test

The paw retraction test was performed by placing each SD rat on a Perspex platform, measuring 30 × 30 cm with a height of 20 cm and with four holes as described previously by Ellenbroek et al., 1987 [31]. The minimum time was reported to be 1 s and a maximum time of 30 s. 

#### 2.2.2. Beam Travel Test

The beam test was performed as previously described by Fleming et al., 2013 [32]. A 1 m long and 25 mm wide inclined beam was used for this experiment. Each SD rat was placed at the lower end of the beam and the time taken for each rat to cross the beam to reach the home cage on the other end was recorded. A maximum time of 60 s was fixed, at the end of which if the rats failed to walk or reach the home cage, the rats received a score of 60 s. 

#### 2.2.3. Cylinder Test

The cylinder test assesses spontaneous activity like rearing and grooming. The test was performed as described by Magno et al., 2019 [33]. Each SD rat was tested for 3 min, and the number of rears made during this period was recorded. This included the full rear where the animal was vertical with the forelimbs off the ground or one or both the forelimbs touching the glass cylinder. 

### 2.3. Surgical Resection and Collection of Brain Sample

At the end of the respective T3 isomers (α- and γ-T3) feeding period (28 days), the SD rats were anaesthetised, by holding them in a desiccator containing diethyl ether, and dissected on a board placed on the ice. An incision was made on the scalp to expose the skull. The skull was removed by breaking it into small pieces with bone-cutting forceps. The brain from each rat was carefully collected in a tube containing 10% neutral-buffered formalin. After 24 h, the brain samples were transferred into a fresh tube containing 10% formalin and were kept immersed in this solution for 24–48 h to fix the tissue.

### 2.4. Tissue Processing 

Following fixation, the brain samples were dissected, and the striatum and substantia nigra areas in the respective brain tissues were isolated. The isolated tissues were placed in an appropriately labelled specimen cassette. Then, the cassettes were loaded into an automated tissue processing machine (Leica, Germany) for dehydration, clearing, and paraffin wax impregnation. The dehydration process involves progressive water replacement in the specimens with increasing ethanol concentration until the tissues are in 100% ethanol. Subsequently, the tissue is cleared with xylene, an intermediate solvent between ethanol and hydrophobic paraffin wax. Specimen infiltrated with paraffin wax were placed in the mould with the desired orientation following the plane of sectioning and embedded with molten paraffin wax to form tissue blocks that were stored at 4 °C until sectioning. A series of 4–5 µm thick sections were cut from each tissue block using a manual rotary microtome (Leica, Germany). The slides were left to dry overnight at room temperature, and the following day, the slides were placed on a hot plate (50 °C) for 5–10 min to allow the paraffin to melt to facilitate the proper adherence of the tissue to the slide.

### 2.5. Immunohistochemistry

Slides with the tissue section was deparaffinised, rehydrated, and subjected to heat-mediated antigen retrieval using the target retrieval solution (Dako, Denmark) at a 1:10 ratio with distilled water, set at 80 °C for 30–40 min. A peroxidase blocking step using a peroxidase blocking solution (Dako, Denmark) was performed at room temperature (RT) for 10 min to reduce non-specific background. 

The tissue sections were incubated with primary antibodies of interest (tyrosine hydroxylase (TH); glial fibrillary acidic protein (GFAP); and neurofilament light chain (NF-L)). The dilution and incubation times for each primary antibody were as per the manufacturer’s (Abcam) protocol ((Anti-GFAP antibody (1:1000, overnight at 4 °C); Anti-TH antibody (1:750, 90 min at RT); Anti-68kDa NF-L antibody (1D2) (1:5000, overnight at 4 °C)). At the end of the incubation period, the slides were washed with TBS. Subsequently, the sections were incubated with species-specific biotinylated secondary antibody (1:500) for 20–30 min. After washing with TBS, the slides were incubated with horseradish peroxidase (HRP) (Dako, Denmark) for 20 min. Once again, the slides were washed with TBS, and incubated with the DAB substrate chromogen solution (1:50 Dako, Denmark) at RT for 20 min. Finally, the slides were washed, and counterstained with Gill’s haematoxylin, dehydrated, and mounted with dibutylphthalate polystyrene xylene (DPX). The immunohistochemistry (IHC) staining in the absence of the primary antibody was carried out as a control for non-specific binding of the secondary antibody. 

### 2.6. Image Analysis

The images of the slides stained with immunomarkers were captured using the Olympus CX21 optical microscope (Olympus, NY, USA) fitted with an eyepiece camera (Dino-Eye Microscope Eyepiece camera). The ImageJ free software was used to quantify the staining intensity of the neuronal fibres and the immunostained cells, as described previously [34]. Immunostained neurons or glial cells in the selected area were counted using the “cell counter” plugin in ImageJ software. The ImageJ software was calibrated before calculating the striatal fibre density to obtain different mean values for the different shades of grey and the matching optical density (OD) for each mean value was calculated. The image to be analysed was first converted to a greyscale image, and the OD of the region of interest (ROI) was measured by subtracting the background OD to determine the final OD of the ROI. 

### 2.7. Statistical Analysis

All the results are presented as mean ± SEM (standard error of the mean). Data from behavioural tests were analysed using the SPSS software package (IBM SPSS Statistic V25). One-way ANOVA with Tukey’s test and multiple comparison were used to compare between the groups and within the groups. The IHC images were analysed using ImageJ software for grey scale analysis followed by one-way ANOVA Tukey’s test. The significance value was set as *p* ≤ 0.05 for all statistical tests.

## 3. Results

### 3.1. Behavioural Evaluations

**Figure 1:**
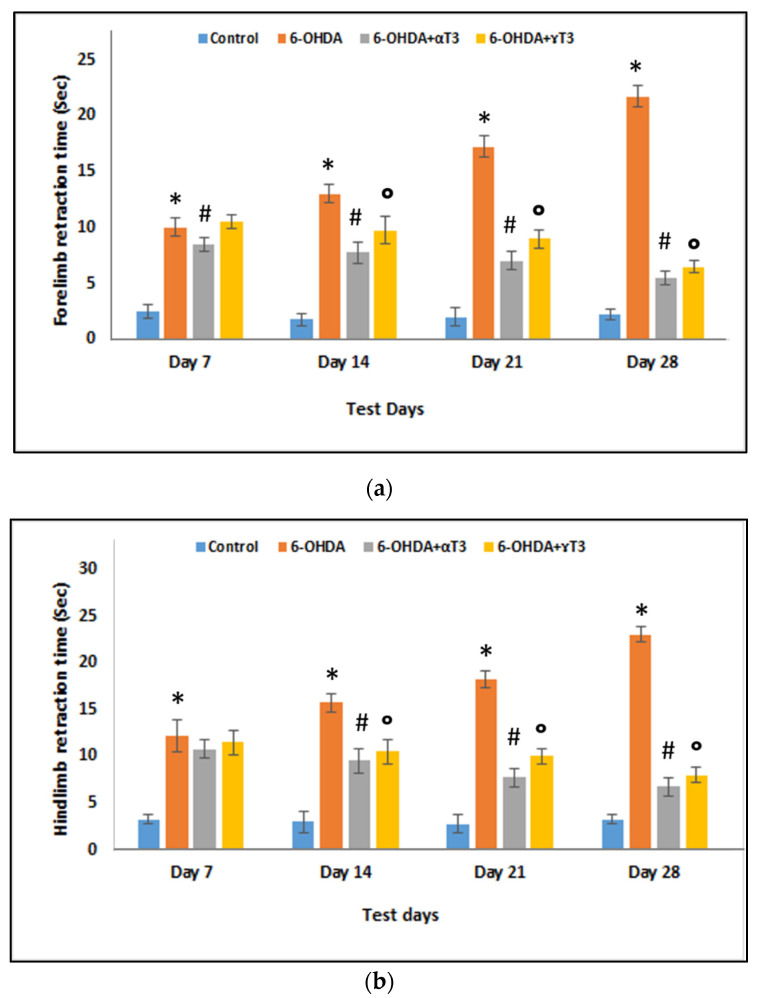
Protective effects of α- and γ-T3 supplementation on (**a**) Forelimb retraction time (FRT) latency and (**b**) Hindlimb retraction time (HRT) latency compared to that in the 250 µg 6-OHDA-induced parkinsonism group (6-OHDA group). (*n* = 6; * *p* < 0.05 vs. control; # *p* < 0.05 vs. 250 µg 6-OHDA; ° *p* < 0.05 vs. 250 µg 6-OHDA).

The rats injected with 250 µg 6-OHDA (6-OHDA group) showed a significant latency in forelimb retraction time (FRT) (Figure 1a) and hindlimb retraction time (HRT) (Figure 1b) on all test days compared to that of the respective control group. The latency of limb retraction (FRT and HRT) in the 6-OHDA group increased gradually as the weeks progressed from day 7 to day 28, indicating a gradual increase in motor impairment due to progressive neurotoxicity. In contrast, supplementation with α-T3 significantly decreased FRT on days 7, 14, 21, and 28, compared to that of the respective 6-OHDA-injected group. The decrease in FRT in the α-T3-supplemented group progressed gradually from day 7 to day 28. However, the rats supplemented with γ-T3 (6-OHDA+ γ-T3) showed a significant decrease in FRT on days 14, 21, and 28 compared to that of the respective 6-OHDA group. Similarly, HRT decreased significantly in rats fed with α-T3 (6-OHDA+α-T3) or γ-T3 (6-OHDA+ γ-T3) on days 14, 21, and 28 compared to that of the respective weeks’ 6-OHDA group. 

**Figure 2:**
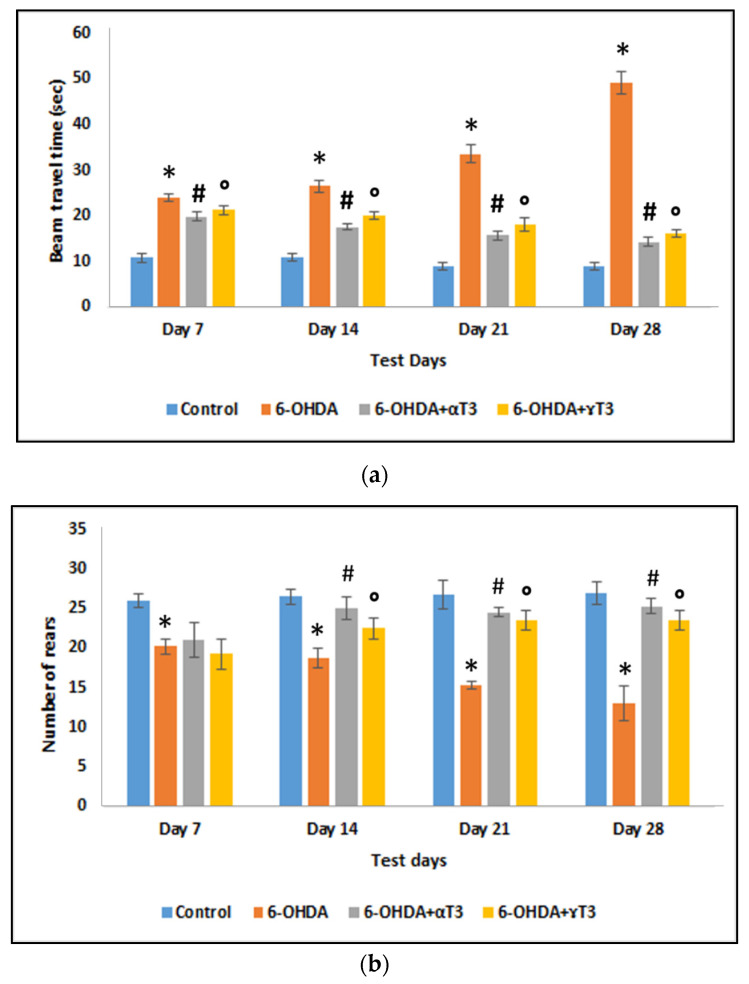
(**a**) Reduced beam travel time and (**b**) increased total number of rears in T3 (α- and γT3) supplemented groups compared to the 6-OHDA-injected group. (*n* = 6; * *p < 0.05* vs. control; # *p* < 0.05 vs. 250 µg 6-OHDA; ° *p* < 0.05 vs. 250 µg 6-OHDA).

The beam travel time (Figure 2a) of SD rats injected with 250 µg 6-OHDA significantly increased on all test days compared to that of the respective control group. The beam travel time gradually increased as the days progressed from day 7 to day 28, indicating motor impairment caused by 6-OHDA neurotoxicity. In contrast, the α-T3 (6-OHDA+α-T3) supplemented groups showed a significant decrease in the beam travel time on all test days compared to that of the 6-OHDA group. Whereas the γ-T3 (6-OHDA+ γ-T3) supplemented group showed a significant decrease in the beam travel time on the test days 14 to 28 compared to the that of the respective 6-OHDA group. The protective effect of T3 supplementation was evident as the beam travel time decreased with each passing week. 

In the cylinder test (Figure 2b), the number of rears significantly decreased in the 6-OHDA-injected group on all test days compared to that of the respective control group. The decrease in the number of rears was progressive from day 7 to day 28, indicating progressive DA neuronal loss. Supplementation with α- and γ-T3 showed a significant increase in the number of rears compared to that of the 6-OHDA-injected group on days 14, 21, and 28. 

### 3.2. Immunohistochemistry 

**Figure 3:**
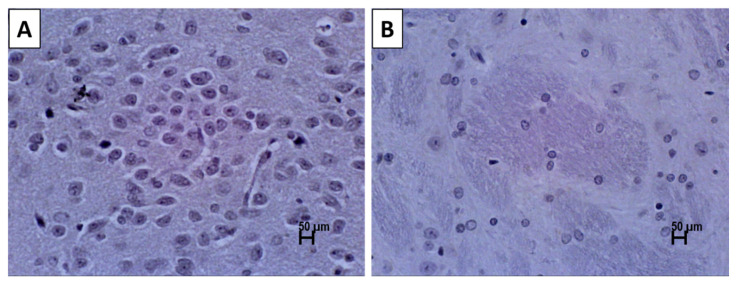
Photomicrographs showing the immunohistochemistry (IHC) without primary antibody for tyrosine hydroxylase (TH) in the coronal sections of (**A**) substantia nigra without detectable dopamine (DA) neurons, and (**B**) striatum without detectable DA fibres. Scale bar: 50 µm.

**Figure 4:**
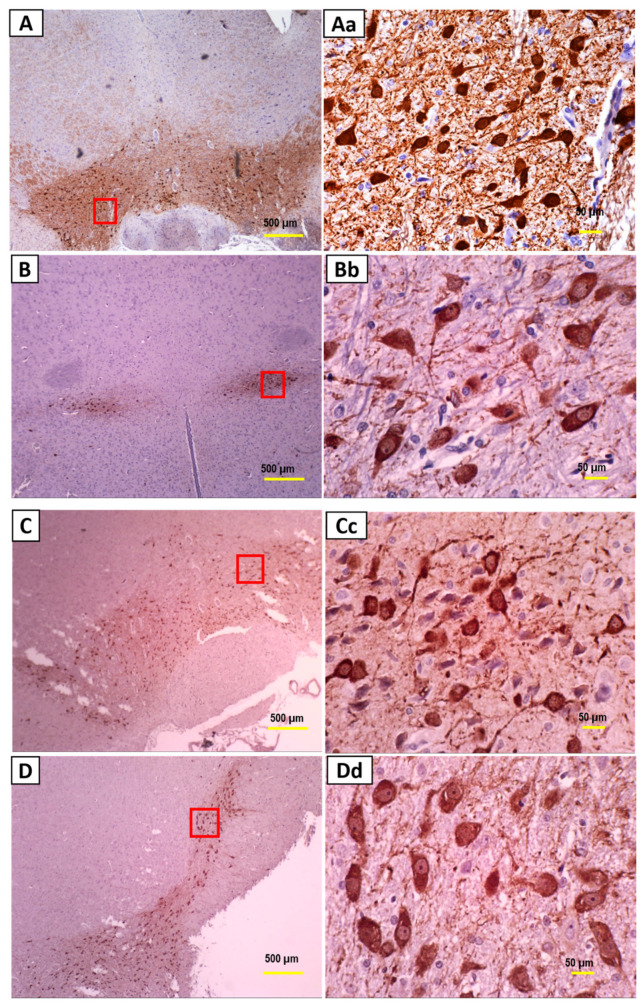
Photomicrographs of tyrosine hydroxylase (TH)-immunostained coronal sections of the substantia nigra (SN) from the (**A**) control group, (**B**) 250 µg 6-OHDA-injected group, (**C**) 250 µg 6-OHDA + α-T3-treated group, and (**D**) 250 µg 6-OHDA + γ-T3-treated group. (Left half (**A**–**D**) Lower magnification, Scale bar: 500 µm; Right half (**Aa**–**Dd**) higher magnification, Scale bar: 50 µm).

**Figure 5:**
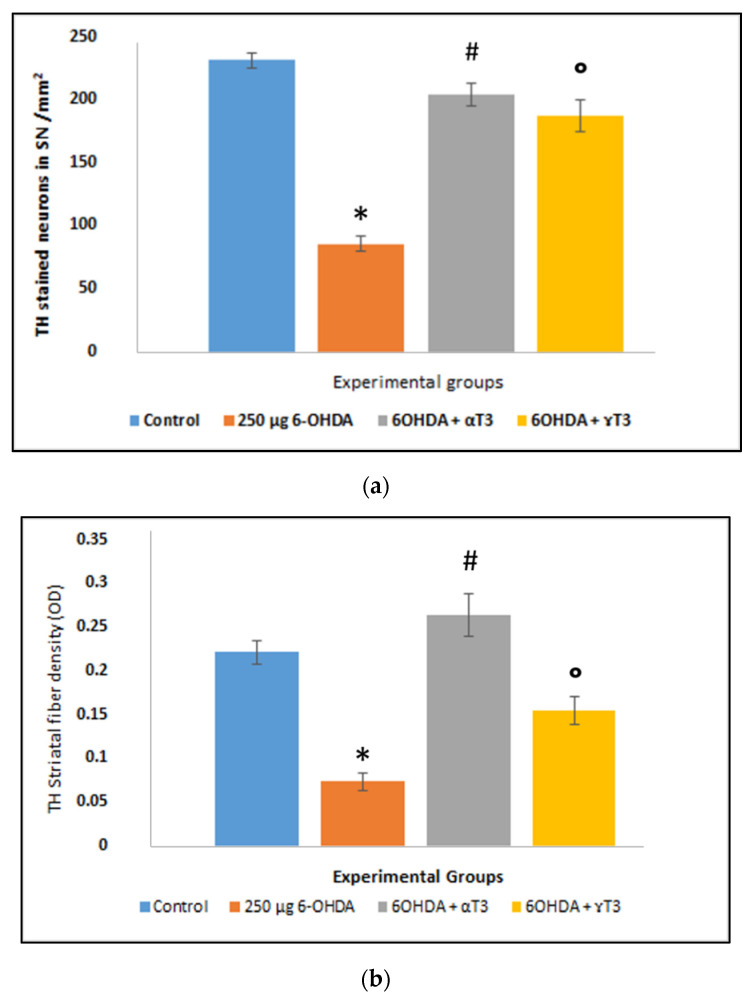
Bar chart representing the (**a**) average number of tyrosine hydroxylase (TH)-immunostained dopamine neurons in the substantia nigra, and (**b**) TH-immunostained striatal fibre density (optical density (OD)) quantified using ImageJ. (*n* = 6; * *p* < 0.05 vs. control; # *p* < 0.05 vs. 250 µg 6-OHDA; ° *p* < 0.05 vs. 250 µg 6-OHDA).

**Figure 6:**
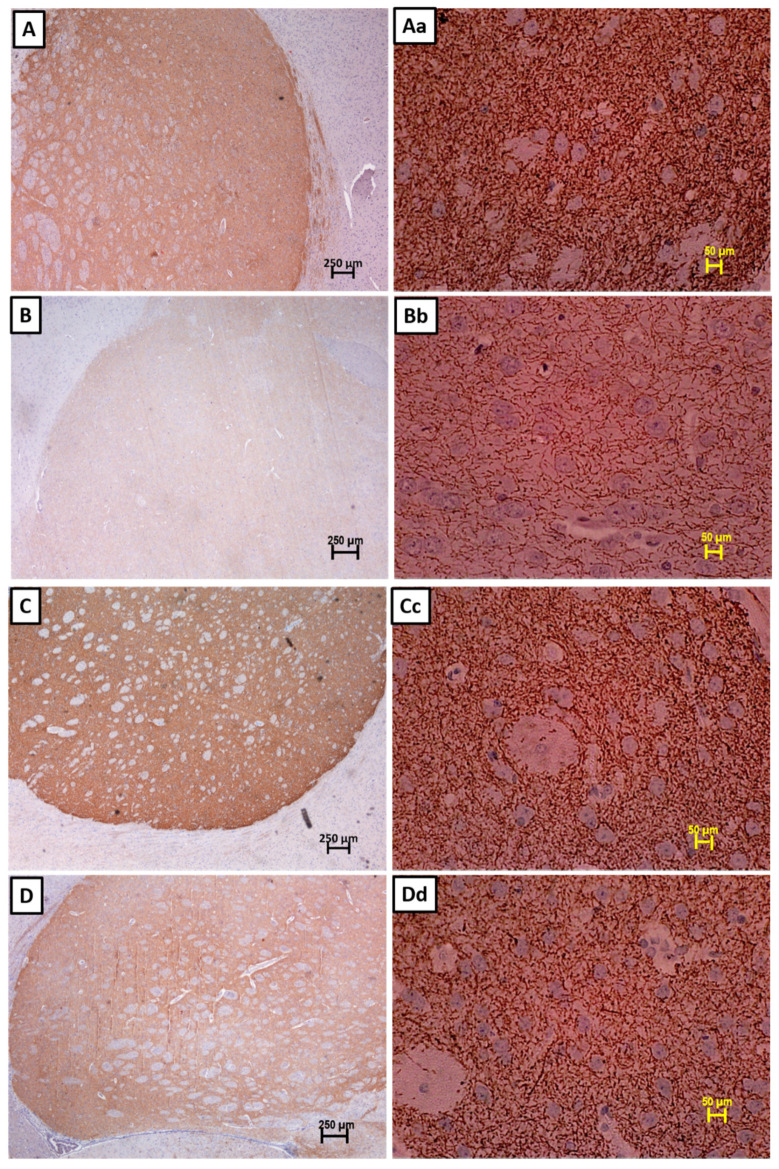
Photomicrographs of tyrosine hydroxylase (TH)-immunostained coronal sections of striatum (STR) from the (**A**) control group showing intense staining and dense fibres, (**B**) 250 µg 6-OHDA-injected group showing weak staining and reduced fibre density due to dopaminergic striatal fibre loss, (**C**) 250 µg 6-OHDA + α-T3-treated group, and (**D**) 250 µg 6-OHDA + γ-T3-treated group showing intense staining and dense fibres due to rescue of DA fibres by both isomers of T3. (Left half (**A**–**D**) lower magnification, Scale bar: 250 µm; Right half (**Aa**–**Dd**) higher magnification, Scale bar: 50 µm).

A control staining was carried out in absence of primary antibodies, to ensure the validity of the staining and to eliminate the possibility of non-specific binding with the secondary antibody. No positive staining was observed when the primary antibody for TH was omitted indicating that there was no false-positive binding of the secondary antibody (Figure 3). The number of TH positive neurons significantly reduced in the SD rats’ brains injected with 250 µg 6-OHDA compared to that of the control group (Figure 4 and Figure 5a). Supplementation with α- and γ-T3 appears to significantly prevent the loss of TH dopamine neurons in the SN. A similar effect was seen in striatal fibre density, which was significantly reduced in the 250 µg 6-OHDA group (Figure 5b and Figure 6) compared to the control group). In the T3 (α- and γ-T3) fed groups there was a significant prevention of loss of striatal fibre density compared to that of the 6-OHDA group. 

**Figure 7:**
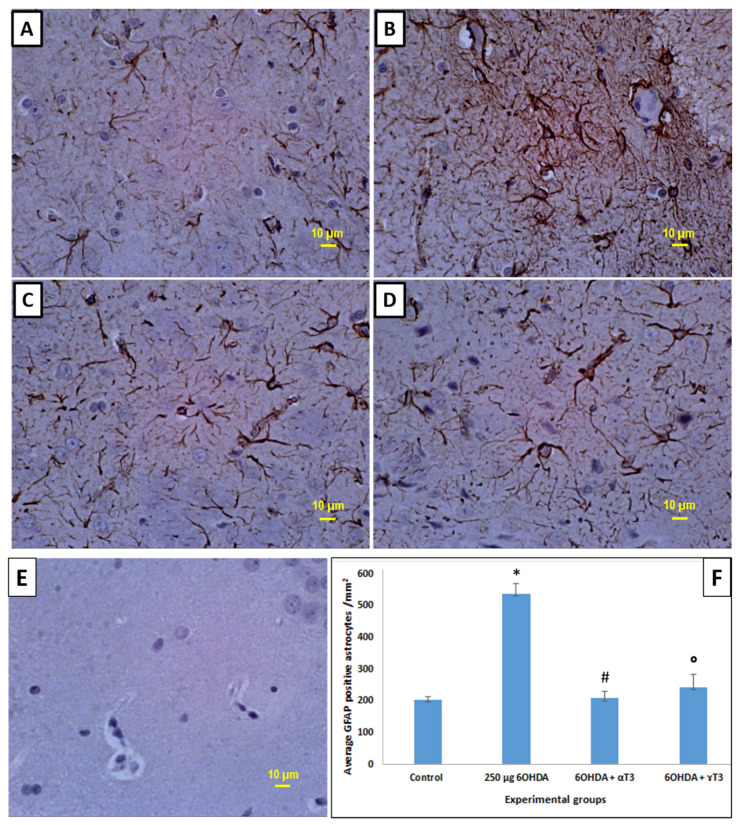
Photomicrographs showing the expression of glial fibrillary acidic protein (GFAP) in striatum (STR) from the (**A**) control group, (**B**) 250 µg 6-OHDA-injected group, (**C**) 250 µg 6-OHDA + α-T3-treated group, (**D**) 250 µg 6-OHDA + γ-T3-treated group, and (**E**) negative control without primary antibody for GFAP and (**F**) bar chart showing the quantification of GFAP positive astrocytes in STR of A–D. (Scale Bar: 10 µm; *n* = 6; * *p* < 0.05 vs. control; # *p* < 0.05 vs. 250 µg 6-OHDA; ° *p* < 0.05 vs. 250 µg 6-OHDA).

Severe astrogliyosis with an increased network of GFAP positive stained processes and glial scars were seen in the 6-OHDA-injected group compared to that of the control group (Figure 7). The number of GFAP positive astrocytes in the 250 µg 6-OHDA-injected group compared to the control group were quantified and found significantly (Figure 7F) increased, suggesting severe neuroinflammation. A reduction in the striatal astrogliosis was observed with supplementation of both α- and γ-T3, with α-T3 showing a more pronounced effect than the γ-T3. However, the reduction was significant in both α- and γ-T3-fed groups compared to that of the control group. 

**Figure 8:**
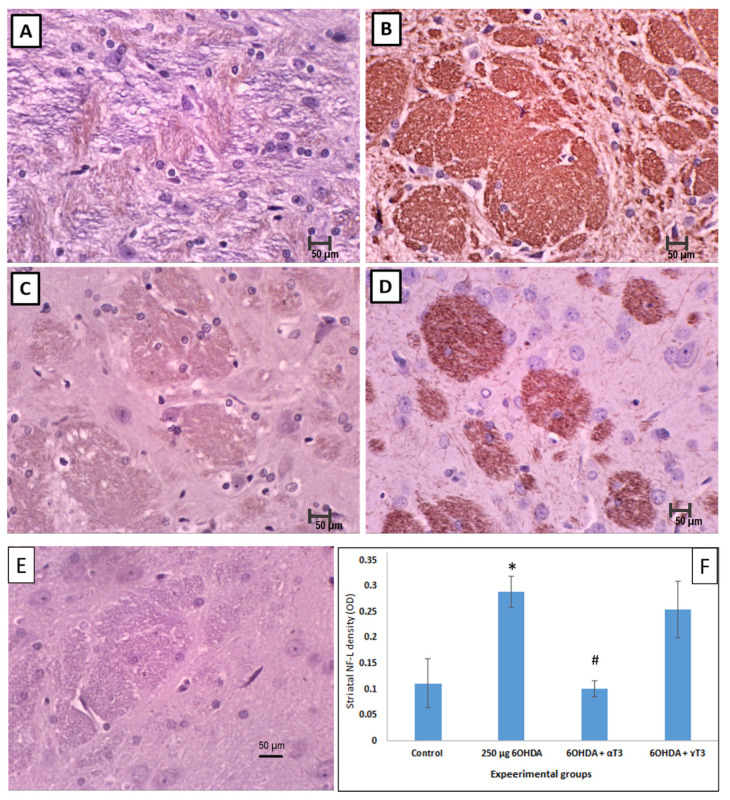
Photomicrographs showing the abnormal accumulation of neurofilament with neurofilament light chain (NF-L) immunostaining in the brains of Sprague Dawley (SD) rats injected with 250 µg 6-OHDA (**B**) compared to that of the control group (**A**). Mitigation of the effects following treatment with α-T3 (**C**) and γ-T3 (**D**) were observed. Negative control staining without primary antibody for NF-L (**E**) and bar chart showing the quantification of NF-L positive fibres in STR of A–D (**F**). (Scale Bar: 50 µm; *n* = 6; * *p* < 0.05 vs. control; # *p* < 0.05 vs. 250 µg 6-OHDA).

There was a marked increase of NF-L expression in the 6-OHDA group compared to that of the control group (Figure 8). In contrast, the NF-L expression was observed to be reduced in the brains of SD rats supplemented with α- or γ-T3, while the former showing a pronounced reduction in NF-L expression. The NF-L fibre density was quantified, and the observation showed that the NF-L fibre density was significantly (Figure 8F) increased in the 6-OHDA-injected group when compared with that of the control group. As for the T3 isomers-supplemented groups, α-T3 was noted to have a significant reduction in the density of the NF-L fibre when compared to that of the 6-OHDA group. 

## 4. Discussion

Parkinson’s disease is a progressive disorder of the central nervous system (CNS) characterised by a varying degree of impaired motor and non-motor features. At present, there are no definitive tests to help in the diagnosis of PD. Hence, clinicians rely on the cardinal clinical features such as motor symptoms as the basis for diagnosing this disease [35]. The neurotoxin 6-OHDA, when injected into animals, causes selective loss of DA neurons in the nigrostriatal pathway; which in turn causes grave motor impairments and reduced movements that are fairly comparable to those observed in late-stage PD patients who have DA loss; thus emulating disease progression in animal models [25]. 

A study conducted on parkinsonian clinical cases reported that the first sign of disease appeared when there was a loss of 50% of neurons in the SN [36]. Other researchers reported about 30% loss of the SN neurons and striatal density when the first motor signs of the disease appeared [37]. In the present study, SD rats injected with 250 µg 6-OHDA closely resemble the disease progression in PD patients. In PD, the paramount importance of any therapeutic strategy should be to induce an increase of activity in the nigrostriatal system, which will reverse the DA neuronal loss and maintain an appropriate concentration of dopamine in the brain [38]. 

The motor behavioural tests are very sensitive and can assess the extent of the nigrostriatal dopaminergic loss. The degree of progressive DA neuronal loss in the nigrostriatal pathway over a period of time is responsible for the decreased level of locomotor activity [39]. In this study, the 6-OHDA-injected SD rats showed decreased locomotion and impaired coordination on the beam travel with each passing week, confirming the progressive DA neuronal loss. The beam travel test analyses skilled walking; hence, increased errors in this function denotes impaired coordination and balance, which is due to the loss of nigrostriatal dopamine [40]. The paw retraction test showed muscular rigidity in 6-OHDA-injected rats and thus prolonged retraction time for both fore and hind paw, suggesting catalepsy induced by 6-OHDA [31]. 

In this study, it is evident that the SD rats with intracisternal 6-OHDA injection exhibited behavioural alterations, such as motor deficits and dyskinesia, similar to clinical features observed in PD patients. We employed the behavioural study and immunohistochemistry analysis of three important markers, TH, GFAP, and NF-L, to evaluate the ability of tocotrienol isomers (α- and γ-T3) to offer neuroprotective effects in the 6-OHDA-lesioned brain of the SD rats. As the behavioural patterns are closely associated to the degree of neuronal dysfunction [41], in our study, we have observed that 6-OHDA injection led to severe motor deficits and dopaminergic neuronal loss in SD rats. In contrast, supplementation with T3 isomers significantly decreased both FRT and HRT and improved the neuronal functions and motor behavioural effects in these animals. The immune-markers TH, GFAP, and NF-L are reliable markers to evaluate nigrostriatal DA neuronal depletion, astrogliosis, and inflammation in 6-OHDA-induced rats. The oral administration of both α- and γ-T3 to 6-OHDA-induced rats showed an increase in TH expression that correlated to the increased DA neurons and striatal fibres as seen in the immunohistochemistry. The precursor for the synthesis of DA is the amino acid tyrosine. The enzyme tyrosine hydroxylase (TH) is the rate-limiting enzyme involved in step one of the sub-pathway that synthesises dopamine (DA) from L-tyrosine. The DA converts tyrosine to L-Dopa (dihydroxyphenylalanine), following which the enzyme L-amino acid decarboxylase (DDC) converts L-dopa to DA [42]. Hence, it is crucial to check the level of TH expression in SN and STR regions of the brain to assess the level of DA loss that is associated with PD pathology. Previous animal models that used 6-OHDA injection to induce neuroinflammation showed death of dopaminergic neurons in the substantia nigra pars compacta based on the decrease in the number of TH positive neurons, which correlated with the behaviour [43]. In the brain, reactive oxygen species (ROS) can cause neuroinflammation that progresses to neuronal cell death through a cascade of events. For instance, activated neuroglia (astrocytes and microglia) lead to the chronic production of pro-inflammatory cytokines that eventually causes DA neuronal death [44]. Astrocytes are the most abundant glial cells of CNS and have crucial roles in maintaining the normal functioning of the CNS, such as maintenance of the blood–brain barrier (BBB), neurotransmitter clearance, homeostatic balance, inflammation, and ionic balance [45]. As such, any insults to the CNS, such as trauma or neurodegenerative diseases, can cause the molecular expression of the astrocytes to change through a process known as astrogliosis, which is in fact a defence mechanism of the astrocytes when encountered with an external stimuli [45]. Similar to this approach, we used 6-OHDA as an external stimulus to insult the CNS. The most common astrocyte cell marker used in neurological studies is GFAP, a class-III intermediate filament that is found in mature and developing astrocytes in the CNS. The GFAP marker is used to investigate the neuroinflammation induced by 6-OHDA whereby activation of the astrocyte population known as astrogliosis was shown to take place. In the present study, the higher expression of GFAP immunoreactivity caused by the 6-OHDA insult to the brain was ameliorated by T3 (α- and γ-T3) supplementation in SD rats. The GFAP-positive cell density reduced by almost 50% in the T3-supplemented groups; more so in the α-T3 group. The neurotoxin 6-OHDA causes disruption of the BBB and neuroinflammation [46], resulting in an activation of glial cells. Irregularity in the functioning of astrocytes was shown to influence the integrity of neurons negatively. A previous study that employed immunofluorescence microscopy revealed that GFAP expression significantly increased in 6-OHDA-induced Wistar rats [43]. Another study also showed that 6-OHDA induced motor deficits, TH loss in the SN, and overactivation of GFAP+ cells in a 6-OHDA-injected rat model. In another study of a 6-OHDA animal model, the increase in the activation of glial cells and subsequent release of pro-inflammatory and anti-inflammatory cytokines was observed at the site of the 6-OHDA injection [47]. In the present study, the higher expression of GFAP immunoreactivity caused by the 6-OHDA insult to the brain was ameliorated by T3 (α- and γ-T3) supplementation in SD rats. The beneficial effects observed could be attributed to the fact that T3 are lipid-soluble vitamins that can penetrate the saturated fatty layers of the BBB (transcellular lipophilic pathway) because of its structural configuration of having an unsaturated side chain [48]. Uptake of both α-T3 and γ-T3 by the hippocampal neuronal cells was shown by Sen et. al., however, α-T3 exhibited a pronounced neuroprotective effect [49].

The beneficial effects of vitamin E supplementation on behavioural aspects were previously demonstrated in a rotenone-induced PD model [50]. However, these authors did not specify the analogue of vitamin E used in their study. The results from the present study strongly suggest that T3 (α- and γ-T3) functionally rescued the DA neurons in SN and STR fibres after 28 days of oral supplementation [43], with α-T3 showing pronounced effects compared to that of γ-T3. Other researchers, who had compared the effects of different vitamin E analogues, also found that α-T3 showed the highest neuroprotection in a cell-based model of striatal neurons [19] and this evidence is in agreement with the findings obtained in our study. In another study, δ-T3 was found to attenuate neurotoxicity and improve motor deficits in a mouse model of PD induced by 1-methyl-4-phenyl-1,2,3,6-tetrahydropyridine (MPTP) [51]. 

Neurofilaments are the structural cytoskeletons of neurons and are highly concentrated in axons compared to their concentration in the cell body or dendrites in most neurons [52]. The neurofilament is composed of three intermediate filament proteins, named L, M, and H. The light subunit (NF-L) is the most abundant and is frequently used as a biomarker for degradation of neurons. NF-L immunoreactivity is frequently used as a biomarker for the degradation of neurons and assesses the severity of neurological deficits. The accumulation of NF-L is positively related to the severity of motor impairments [53]. Increased NF-L levels in the cerebrospinal fluid is associated with severe motor deficits, non-motor symptoms such as anosmia, and decreased survival rate as stated in a cohort study [53]. The present study showed increased accumulation of NF-L in the brains of SD rats injected with 6-OHDA when compared to that of the control group. The accumulation of NF-L in the 6-OHDA-injected animal group correlates with the parkinsonism motor symptoms, similar to the previous study [53]. Supplementation with T3 (α- and γ-T3) decreased the NF-L expression, thus implying the rescuing of the neurons from degradation. The reduction in the number of neurofilaments in the T3 (α- or γ-T3) supplemented group postulates the improvement and reduction in the severity of the motor function [54]. In line with this, the present study confirms the efficacy of T3 supplementation, which shows improvement in the spontaneous activity of the limb use by suppressing the nigrostriatal pathway damage caused by 6-OHDA neurotoxicity. Overall, in all the expressional studies, α-T3 showed better neuroprotective effects than did γ-T3. 

## 5. Conclusions

The results from this study revealed that the supplementation with tocotrienols (α- or γ-T3) alleviated the PD-like symptoms observed in terms of improvement in motor behaviour of SD rats, which is positively correlated to DA neuronal health, reduced astroglial activation, and reduced accumulation of neurofilaments. Although both α- and γ-T3 demonstrated neuroprotective effects, α-T3 had significantly higher effects compared to those of γ-T3 in this experimental model. Considering that oxidative stress is a major issue in PD, the results strongly support the potential antioxidant role of T3 against neurodegeneration, which may play a crucial role in reducing neuroinflammation. These findings strongly suggest that supplementation with tocotrienol has a therapeutic potential in the treatment of PD. Further studies exploring the different doses and durations of tocotrienols in PD models are required to understand the molecular mechanism of its neuroprotective effects.
